# Survival outcome and impact of delayed imatinib therapy in gastric gastrointestinal stromal tumors

**DOI:** 10.3389/fsurg.2025.1569677

**Published:** 2025-04-03

**Authors:** R. Jansuwan, S. Samphao, Wongsakorn Chaochankit

**Affiliations:** Department of Surgery, Faculty of Medicine, Prince of Songkla University, Songkhla, Thailand

**Keywords:** gastrointestinal stromal tumor, gastric GIST, disease-free survival, imatinib therapy, delayed treatment, prognostic factors

## Abstract

**Background:**

Gastrointestinal stromal tumors (GISTs) are the most common mesenchymal tumors of the gastrointestinal tract, with the stomach being the predominant site. Surgical resection is the primary treatment for localized disease, but recurrence remains a concern, particularly in high-risk patients. Tyrosine kinase inhibitors (TKIs), such as imatinib, improve disease-free survival (DFS), yet their accessibility is often limited in resource-constrained settings.

**Methods:**

This retrospective cohort study included gastric GIST patients who underwent surgical resection between 2015 and 2020 at a tertiary referral center. DFS and overall survival (OS) were analyzed using Kaplan–Meier curves and Cox proportional hazards regression.

**Results:**

A total of 86 patients were included, with 40 (46%) classified as high-risk. The 5-year DFS was significantly lower in high-risk patients (40% vs. 95.7%, *p* < 0.001). Imatinib therapy group was associated with worse DFS in high-risk patients (*p* = 0.003), likely due to delayed initiation after recurrence rather than adjuvant use. Significant predictors of poor DFS included smoking (*p* < 0.001), prolonged operative time (*p* = 0.034), and advanced tumor stage (*p* < 0.001).

**Conclusion:**

Delayed imatinib therapy negatively impacts DFS in high-risk gastric GIST patients, highlighting the need for improved access to early TKI treatment. Additionally, smoking cessation and optimized perioperative management may enhance survival outcomes. Addressing modifiable risk factors and ensuring timely posoperative treatment could improve prognosis in this population.

## Introduction

Gastrointestinal stromal tumors (GISTs) are the most common mesenchymal tumors of the digestive tract, originating from the interstitial cells of Cajal. These tumors can present as incidental findings or symptomatic lesions, with symptoms such as gastrointestinal bleeding, abdominal pain, early satiety, or palpable masses. In the United States, the incidence of GIST is estimated to be 4,000–6,000 cases annually (1). The prognosis of GISTs is influenced by multiple factors, including tumor size, mitotic rate, organ involvement, and tumor perforation. Among the different anatomical locations, the stomach is the most frequently affected site, accounting for approximately 60% of cases (2).

The primary treatment for localized GIST is surgical resection with the goal of achieving R0 resection, defined as complete tumor removal with negative microscopic margins (3). Studies have shown that the 5-year survival rate in patients with completely resected localized tumors is approximately 54% (4). However, despite successful surgical resection, recurrence remains a significant challenge, particularly in intermediate- and high-risk patients, negatively affecting long-term survival rates. To address this issue, adjuvant therapy with tyrosine kinase inhibitors (TKIs), such as imatinib, has been introduced as an important treatment strategy (5). Imatinib selectively inhibits the KIT and PDGFRA mutations, which are key drivers of GIST pathogenesis, thereby reducing the likelihood of recurrence and improving overall survival (5).

Despite the established benefits of imatinib in improving disease-free survival (DFS) and overall survival (OS), access to this medication remains a major challenge in developing countries. In Thailand, as in many other resource-limited settings, the reimbursement policies restrict the use of imatinib primarily to patients with advanced-stage disease or those with tumor rupture. As a result, patients with intermediate- or high-risk tumors who might benefit from adjuvant imatinib often do not receive it, which could negatively impact their survival outcomes (6, 7).

Several studies have demonstrated that adjuvant imatinib significantly prolongs DFS, particularly in high-risk GIST patients (8). However, in settings where the use of imatinib is limited to only advanced-stage cases, its full potential in reducing recurrence and improving outcomes remains underexplored (9). Investigating the survival outcomes of patients who received imatinib vs. those who did not, within our specific healthcare system, could provide valuable insights into the importance of expanding access to TKIs for patients at risk of recurrence (10).

Given these challenges, this study aims to evaluate the DFS rates of patients with gastric GISTs at Songklanagarind Hospital and identify the clinical, pathological, and surgical factors associated with disease progression. A particular focus will be placed on understanding the impact of imatinib use within our healthcare setting, where access to the drug is limited by reimbursement policies. By analyzing the survival outcomes of both treated and untreated patients, we hope to provide evidence that may support broader access to TKIs in intermediate- and high-risk patients, ultimately improving long-term outcomes for individuals diagnosed with gastric GISTs. The study aimed to assess survival differences between patients who received adjuvant imatinib and those who did not. This analysis provides critical insights into the impact of treatment accessibility on oncologic outcomes and may inform future healthcare policy adaptations in resource-limited settings.

## Materials and methods

### Study design and population

This retrospective cohort study was conducted at Songklanagarind Hospital, a tertiary referral center in Thailand, to evaluate DFS in patients diagnosed with gastric gastrointestinal stromal tumors (GISTs). Eligible patients included those with histopathologically confirmed gastric GISTs who underwent surgical resection between January 1, 2015, and December 31, 2020. Ethical approval was granted by the Human Research Ethics Committee of the Faculty of Medicine, Prince of Songkla University (project number REC.64-579-10-4).

Patients were classified into two groups based on their recurrence risk: the high-risk group, defined according to the Modified National Institutes of Health (NIH) Consensus Criteria or the presence of metastatic disease at diagnosis, and the non-high-risk group, which included patients with low-to-intermediate risk. Exclusion criteria encompassed patients with concurrent malignancies, a diagnostic age under 18 years, or insufficient follow-up data.

### Data collection and variables

Data were extracted from electronic medical records, including patient demographics, clinical presentation, tumor characteristics, surgical details, pathological findings, and adjuvant therapy. The key variables analyzed included:

• Baseline characteristics: age, sex, history of smoking and alcohol consumption, presenting symptoms (e.g., gastrointestinal bleeding, abdominal mass, early satiety), clinical tumor size, and staging at diagnosis.
• Surgical details: operative approach (open vs. laparoscopic), surgical duration, estimated blood loss, tumor perforation status, and resection margin status (R0, R1, or R2).
  ○ Surgical Approach and Resection Volume: The choice of open vs. laparoscopic surgery was based on tumor size, location, and surgical feasibility. Laparoscopic surgery was preferred for tumors ≤5 cm located in accessible regions (e.g., anterior wall, greater curvature), while open surgery was performed for larger tumors (>5 cm), lesions in difficult locations (e.g., gastroesophageal junction), or those suspected of infiltration into adjacent structures. Resection volume varied depending on tumor location, with most cases undergoing wedge or partial gastrectomy, and total gastrectomy reserved for extensive disease.
• **Pathological findings**: tumor size, mitotic rate [per 50 high-power fields (HPF)], and risk categorization per the Modified NIH criteria.
  ○ Risk Classification: Patients were categorized based on the Modified National Institutes of Health (NIH) Consensus Criteria, which considers tumor size, mitotic rate (per 50 HPF), tumor location, and presence of tumor rupture as key prognostic factors. High-risk patients were defined as those with tumors ≥10 cm, mitotic index >5/50 HPF, non-gastric location, or tumor rupture.
• Adjuvant therapy and outcomes: Patients received imatinib either as postoperative adjuvant therapy, after recurrence, or in rare cases as preoperative therapy. Among high-risk patients, imatinib was primarily used as adjuvant therapy, while in the non-high-risk group, only a small subset of patients received imatinib at the clinician's discretion, likely due to borderline risk classification or individual patient factors. Preoperative imatinib use was reserved for patients with locally advanced or borderline-resectable tumors. DFS (time from surgery to recurrence), progression-free survival (PFS, time from disease progression in advanced stage disease), and overall survival (OS).

### Statistical analysis

Continuous variables were summarized as medians with interquartile ranges (IQR) or means with standard deviations (SD), depending on data distribution. Categorical variables were expressed as frequencies and percentages. The independent *t*-test and Mann–Whitney *U*-test were applied for continuous variables, whereas the chi-square or Fisher's exact test was used for categorical variables.

Survival analysis was performed using the Kaplan–Meier method, with DFS and PFS curves compared using the log-rank test including resection margin status (R0 vs. R1/2). Cox proportional hazards regression models were employed to identify independent predictors of DFS. Variables with a *p*-value <0.1 in univariate analysis and those of clinical relevance were included in multivariate models to adjust for potential confounders. Variance inflation factors (VIFs) were assessed to prevent multicollinearity, and statistical significance was set at *p* < 0.05.

## Results

### Study population and baseline characteristics

**Figure 1:**
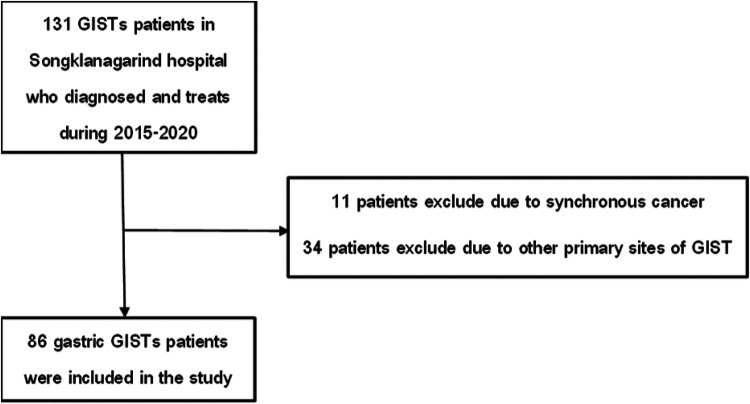
Flow chart of patients with gastric GIST enrolled in the study.

A total of 86 patients diagnosed with gastric GIST were included in the study. Figure 1 illustrates the patient enrollment process, including inclusion and exclusion criteria. Among the initially screened 97 patients, 11 were excluded due to synchronous malignancies, resulting in 86 eligible participants for analysis. Of these, 40 patients (46%) were classified as high-risk, while 46 patients (54%) were categorized as non-high-risk based on the Modified NIH Consensus Criteria. The mean age of the study population was 61.7 years (SD 11.5), with no significant age difference between risk groups (*p* = 0.926). More than half of the participants (54.7%) were female. The most frequently reported presenting symptoms were early satiety or vomiting (38.4%), followed by gastrointestinal bleeding (34.9%) and an abdominal mass (10.5%). Patients in the high-risk group were significantly more likely to present with early satiety or vomiting compared to those in the non-high-risk group (52.5% vs. 26.1%, *p* = 0.005) (Table 1).

**Table 1:** Patients’ baseline characteristics (*N* = 86).

Variables	Total (*n* = 86)	High risk (*n* = 40)	Non-high risk (*n* = 46)	*P*-value
Age (years, SD)	61.7 (11.5)	61.8 (12.2)	61.6 (11.1)	0.926
Female (*n*, %)	47 (54.7)	19 (47.5)	28 (60.9)	0.305
Chief complaint (*n*, %)				
Gastrointestinal bleeding	30 (34.9)	11 (27.5)	19 (41.3)	0.005
Abdominal mass	9 (10.5)	6 (15)	3 (6.5)	
Early satiety, vomiting	33 (38.4)	21 (52.5)	12 (26.1)	
Accidental findings	14 (16.3)	2 (5)	12 (26.1)	
Smoking (*n*, %)	16 (18.6)	11 (27.5)	5 (10.9)	0.089
Alcoholic drinking (*n*, %)	9 (10.5)	7 (17.5)	2 (4.3)	0.075
Clinical tumor size (cm, IQR)	7 (4.2, 12)	11.8 (9, 15.4)	4.5 (3, 6.4)	<0.001
Stage (*n*, %)				
I	44 (51.2)	3 (7.5)	41 (89.1)	<0.001
II	10 (11.6)	6 (15)	4 (8.7)	
III	26 (30.2)	25 (62.5)	1 (2.2)	
IV	6 (7)	6 (15)	0 (0)	
Tissue diagnosis (*n*, %)	24 (27.9)	14 (35)	10 (21.7)	0.260
Preoperative imatinib (*n*, %)	5 (5.8)	2 (5)	3 (6.5)	1.000

SD, standard deviation; IQR, inter quartile range.

### Surgical and pathological outcomes

The majority of patients (91.9%) underwent elective surgery. The open surgery was more frequently performed in the high-risk group (95% vs. 58.7%, *p* < 0.001). Patients classified as high-risk had significantly longer operative times (median 272.5 vs. 180 min, *p* < 0.001) and greater estimated blood loss (median 325 vs. 50 ml, *p* < 0.001). Intraoperative tumor perforation was more common in the high-risk group (15% vs. 2.2%, *p* = 0.046). R0 resection was achieved in 88.4% of cases, with significantly fewer high-risk patients achieving R0 resection compared to the non-high-risk group (80% vs. 95.7%, *p* = 0.006). High-risk patients exhibited significantly larger tumors (median 14.5 cm vs. 4.9 cm, *p* < 0.001) and a higher mitotic rate (median 11 vs. 1 per 50 HPF, *p* < 0.001). Postoperative complications occurred in 10.5% of patients, with a higher frequency in the high-risk group (17.5% vs. 4.3%, *p* = 0.075) (Table 2).

**Table 2:** Surgical details, pathological reports, and outcomes (*N* = 86).

Variables	Total (*n* = 86)	High risk (*n* = 40)	Non high risk (*n* = 46)	*P*-value
Elective surgery (*n*, %)	79 (91.9)	35 (87.5)	44 (95.7)	0.243
Surgical time (min, IQR)	222.5 (176.2, 300)	272.5 (211.2, 349)	180 (140, 240)	<0.001
Estimate blood loss (ml, IQR)	100 (35, 500)	325 (150, 825)	50 (20, 100)	<0.001
Open surgery (*n*, %)	65 (75.6)	38 (95)	27 (58.7)	<0.001
Intraoperative perforation (*n*, %)	7 (8.1)	6 (15)	1 (2.2)	0.046
Resection (*n*, %)				
R0	76 (88.4)	32 (80)	44 (95.7)	0.006
R1	3 (3.5)	1 (2.5)	2 (4.3)	
R2	7 (8.1)	7 (17.5)	0 (0)	
Pathological tumor size (cm, IQR)	7 (4.5, 13)	14.5 (9.8, 18.2)	4.9 (2.8, 6.8)	<0.001
Pathological mitosis (/50 HPF, IQR)	4 (1, 10)	11 (6.8, 25.2)	1 (0, 3)	<0.001
Length of stay (day, IQR)	9 (7, 11.8)	10 (8, 14)	8 (6.2, 9.8)	0.016
Post-operative complication (*n*, %)	9 (10.5)	7 (17.5)	2 (4.3)	0.075
Exposed imatinib (*n*, %)	31 (36)	27 (67.5)	4 (8.7)	<0.001
Post operative imatinib (*n*, %)	30 (34.9)	26 (65)	4 (8.7)	<0.001
Duration of post operative imatinib (month, IQR)	35.5 (20.4, 49.8)	655 (0, 1,148)	0 (0, 0)	<0.001
Total duration of imatinib (month, IQR)	36.6 (22.9, 56.7)	742 (0, 1,185.8)	0 (0, 0)	<0.001
Local recurrence (*n*, %)	8 (9.3)	7 (17.5)	1 (2.2)	0.023
Systemic recurrence (*n*, %)	24 (27.9)	23 (57.5)	1 (2.2)	<0.001
5-year DFS (*n*, %)	58 (71.6)	14 (40)	44 (95.7)	<0.001
Median DFS (month, IQR)	36 (18, 74)	22 (13, 37)	57 (24.5, 104.5)	0.004
5-year DPS (*n*, %)	12 (14)	12 (30)	0 (0)	<0.001
Mortality (*n*, %)	26 (30.2)	23 (57.5)	3 (6.5)	<0.001
Median OS (month, SD)	78.6 (50.3)	85.1 (51.1)	73 (49.4)	0.267

IQR, inter quartile range; HPF, high power field; DFS, disease-free survival; DPS, disease progression survival; OS, overall survival.

### Survival outcomes and prognostic factors

**Figure 2:**
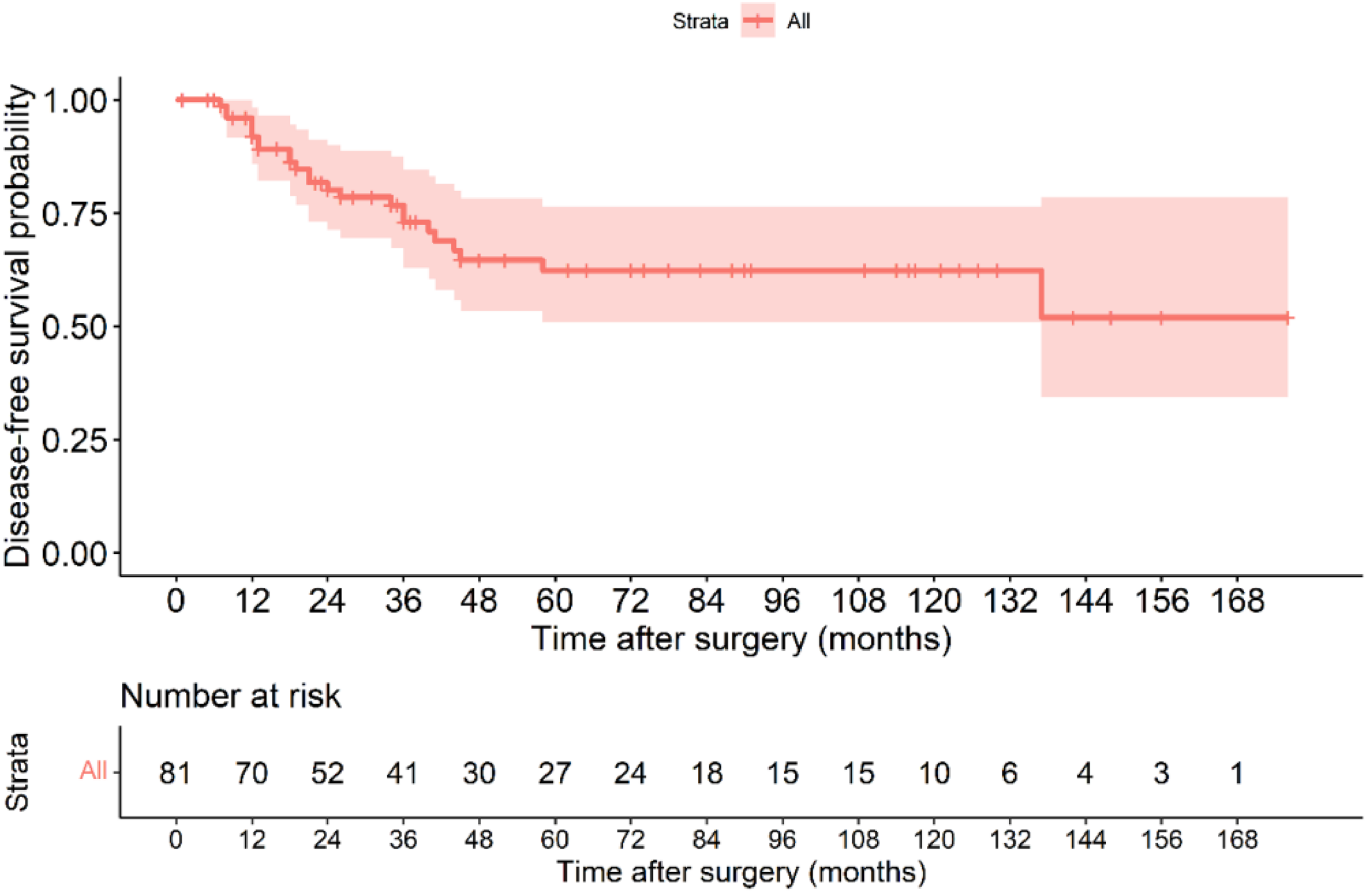
Kaplan–Meier disease-free survival curve of all patients with gastric GIST.

**Figure 3:**
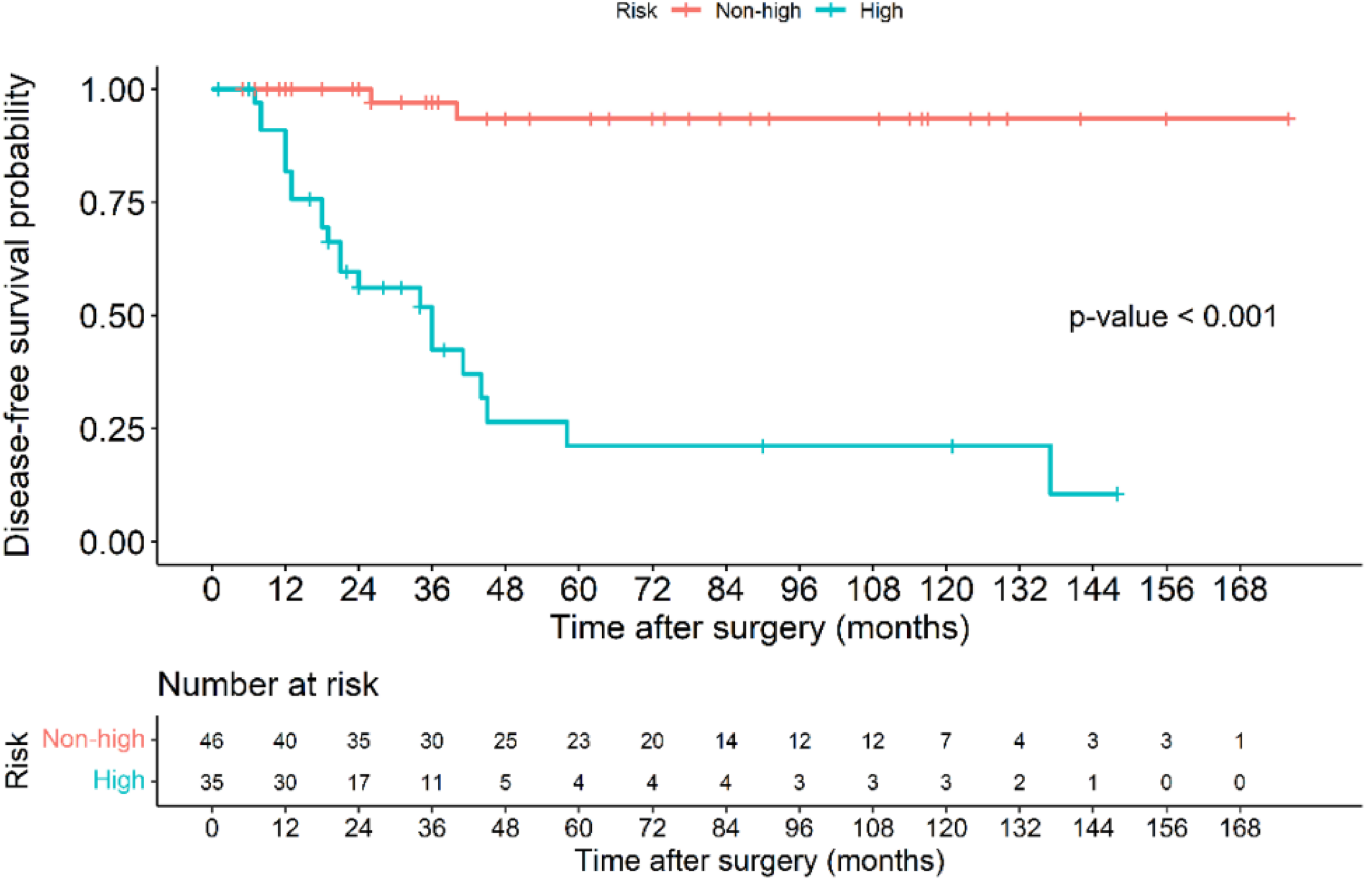
Kaplan–Meier disease-free survival curve of patients with gastric GIST classified based on those in the high-risk and non-high-risk groups.

**Figure 4:**
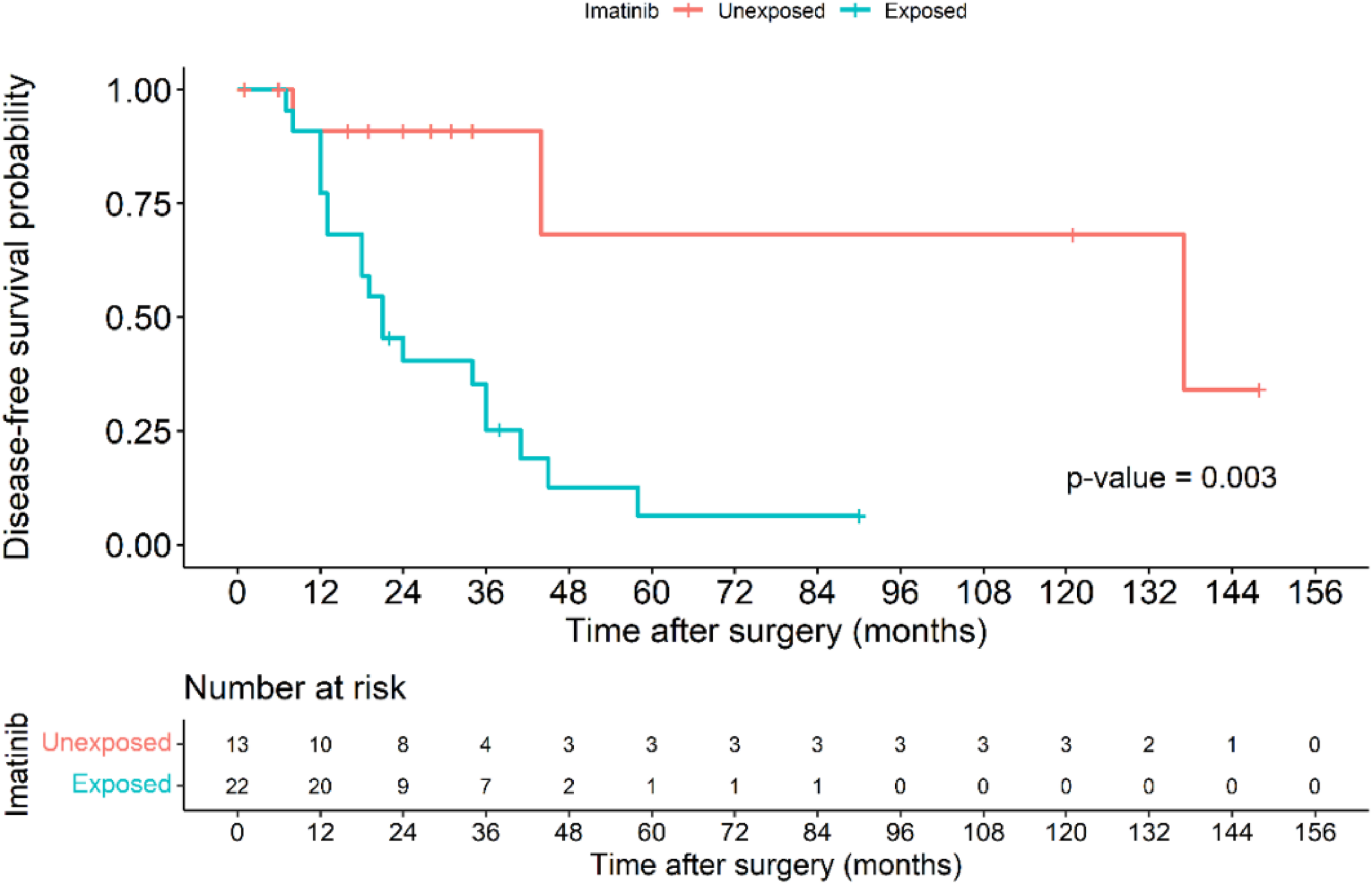
Kaplan–Meier disease-free survival curve of patients in the high-risk group based on those exposure to imatinib and those without imatinib exposure.

The Kaplan–Meier disease-free survival curve (Figure 2) illustrates the probability of remaining recurrence-free over time. The curve shows a gradual decline in DFS, with a pronounced drop observed among high-risk patients, suggesting a strong correlation between tumor aggressiveness and early recurrence. The survival probability significantly declines within the first two years for high-risk patients, whereas non-high-risk patients maintain a relatively stable DFS throughout the follow-up period. The 5-year DFS rate for all patients was 71.6%. The Figure 3 further stratifies DFS by risk groups, highlighting the pronounced disparity in survival outcomes between high-risk and non-high-risk patients. The Kaplan–Meier curve distinctly illustrates that high-risk patients exhibit a steep decline in DFS within the first 36 months post-surgery, whereas non-high-risk patients maintain high survival probabilities throughout the study period. This substantial DFS gap underscores the necessity of early risk stratification and the potential role of intensified postoperative management strategies, including adjuvant therapy, in high-risk patients. A significant difference in DFS was observed between the high-risk and non-high-risk groups (40% vs. 95.7%, *p* < 0.001). In the Figure 4, it shows the impact of imatinib therapy among high-risk patients. The Kaplan–Meier curve demonstrates that high-risk patients who received imatinib exhibited significantly worse DFS compared to those who did not (*p* = 0.003). The decline in DFS was more pronounced in the imatinib-treated group, particularly within the first two years postoperatively. This finding contrasts with previous studies that have demonstrated a beneficial effect of imatinib in preventing recurrence. Among high-risk patients, those who received imatinib had significantly worse DFS compared to those who did not (*p* = 0.003) (Figure 4).

**Figure 5:**
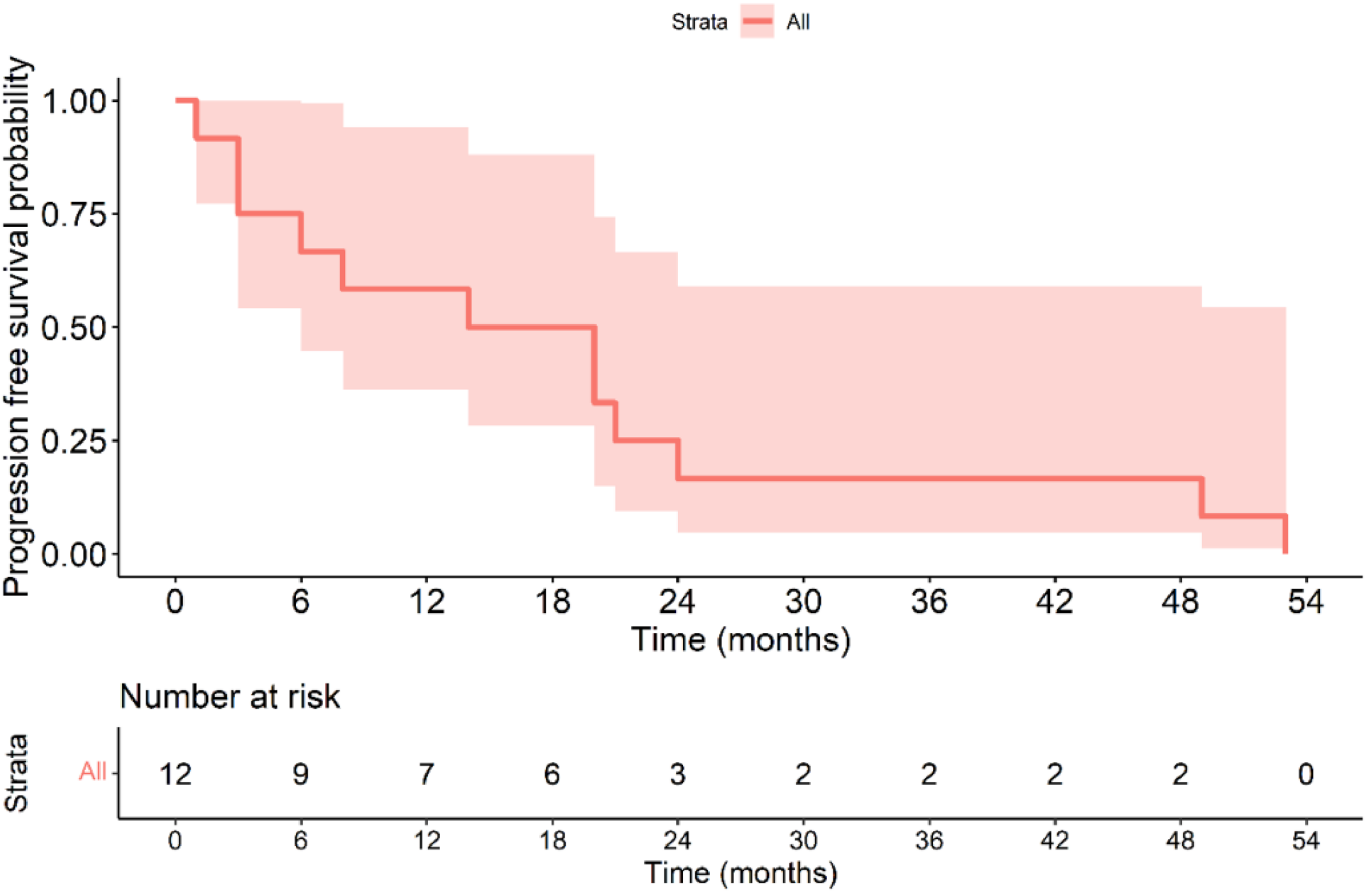
Kaplan–Meier disease progression survival curve of patients with gastric GIST.

In the Figure 5, the Kaplan–Meier disease progression survival (DPS) curve illustrates the probability of patients remaining free from disease progression after recurrence or metastasis. The overall 5-year DPS rate was 14%, with a significantly higher progression rate in the high-risk group (30%) compared to the non-high-risk group (0%, *p* < 0.001). This finding underscores the aggressive nature of high-risk tumors and their rapid progression following recurrence. The curve further indicates that within two years of disease recurrence, the likelihood of further progression sharply increases in high-risk patients, emphasizing the necessity of close monitoring and aggressive intervention strategies. These findings suggest that for patients experiencing recurrence, timely and effective treatment strategies, including systemic therapy and potential surgical interventions, may be essential to delaying disease progression and improving outcomes. Table 3 presents the univariate analysis results identifying factors associated with mortality in patients with gastric GIST. High tumor stage (III–IV) was strongly associated with increased mortality risk, with stage IV patients exhibiting an HR of 20.00 (*p* = 0.03). Tumor size ≥10 cm was also a significant predictor of mortality (HR 5.67, *p* < 0.01), emphasizing the impact of tumor burden on patient survival. Additionally, a pathological mitotic rate of >5/50 HPF demonstrated a strong correlation with mortality (HR 10.00, *p* < 0.001), indicating that increased tumor proliferation significantly worsens prognosis. Other factors linked to increased mortality included R2 resection (HR 7.00, *p* = 0.026), intraoperative tumor perforation (HR 0.36, *p* = 0.013), and estimated blood loss >500 ml (HR 3.82, *p* = 0.009). Moreover, systemic recurrence showed the highest association with mortality (HR 7.73, *p* < 0.001), highlighting the severe impact of metastatic disease progression on overall survival. Interestingly, exposure to imatinib was also associated with increased mortality (HR 6.4, *p* < 0.001), which could reflect the late initiation of therapy in cases with advanced disease rather than a direct adverse effect of the drug. Table 4 presents the results of the multivariate analysis identifying independent predictors of mortality in gastric GIST patients. The high-risk category was the most significant predictor of mortality (HR 27.67, *p* < 0.001), indicating that aggressive tumor characteristics and advanced-stage disease markedly reduce survival outcomes. Interestingly, intraoperative tumor perforation was associated with a lower hazard ratio for mortality (HR 0.05, *p* = 0.026), possibly due to increased postoperative surveillance and subsequent treatment interventions in these patients. Additionally, exposure to imatinib was not significantly associated with mortality in the multivariate model (HR 3.15, *p* = 0.125), suggesting that imatinib's effect on survival may be more nuanced and dependent on treatment timing and tumor response. Table 5 outlines the multivariate analysis results for factors predicting disease-free survival (DFS). Patients aged ≥60 years demonstrated a significantly lower risk of disease recurrence (HR 0.01, *p* < 0.001), which contrasts with some previous studies and may be influenced by selection bias or differences in treatment approaches. Smoking history was strongly associated with worse DFS (HR 706.18, *p* < 0.001), emphasizing the role of lifestyle factors in tumor progression. Tumor staging was another critical determinant, with stage IV patients experiencing the highest risk of recurrence (HR 2,377.18, *p* < 0.001). Furthermore, prolonged operative time (≥200 min) was linked to worse DFS (HR 13.15, *p* = 0.034), suggesting that complex surgical cases may contribute to poorer outcomes due to increased surgical morbidity and tumor manipulation.

**Table 3:** Univariate analysis between mortality and variables in patients with gastric GISTs.

Variables	Crude OR (95% CI)	*P*-value
Age ≥60 years old	1.84 (0.69, 4.88)	0.220
Female	0.61 (0.24, 1.55)	0.299
Chief complaint		
Gastrointestinal bleeding	0.73 (0.15, 3.62)	0.704
Abdominal mass	7.33 (1.11, 48.26)	0.038
Early satiety, vomiting	2.1 (0.49, 9.03)	0.321
Accidental findings	Ref.	
Smoking	2.09 (0.68, 6.39)	0.197
Alcoholic drinking	2.00 (0.49, 8.15)	0.333
Clinical tumor size ≥10 cm	5.67 (2.09, 15.36)	<0.01
Stage		
I	Ref.	
II	4.29 (0.78, 23.43)	0.093
III	13.64 (3.76, 49.49)	<0.001
IV	20 (2.75, 145.48)	0.03
History of biopsy	2.05 (0.76, 5.53)	0.155
Preoperative imatinib	3.78 (0.59, 24.13)	0.159
Operative time ≥200 min	2.92 (1.03, 8.28)	0.044
Estimated blood loss ≥500 ml	3.82 (1.39, 10.49)	0.009
Intraoperative tumor perforation	0.36 (0.04, 3.15)	0.013
Pathological tumor size ≥10 cm	6.21 (2.27, 16.96)	<0.001
Pathological mitotic rate >5/50HPF	10 (3.38, 29.55)	<0.001
High risk category	19.39 (5.14, 73.15)	<0.001
Resection		
R0	Ref.	
R1	1.4 (0.12, 16.29)	0.788
R2	7 (1.26, 38.99)	0.026
Length of stay >7 days	0.96 (0.36, 2.62)	0.943
Postoperative complications	1.17 (0.27, 5.10)	0.831
Postoperative imatinib	4.67 (1.72, 12.65)	0.001
Duration of imatinib >3 years	0.67 (0.16, 2.77)	0.577
Exposed imatinib	6.4 (2.33, 17.61)	<0.001
Local recurrence	2.55 (0.58, 11.08)	0.213
Systemic recurrence	7.73 (2.7, 22.13)	<0.001

OR; odd ratio; Ref, reference; HPF, high power field.

**Table 4:** Determination of factors predicting mortality in patients with gastric GIST using a backward stepwise logistic model.

Variables	Adj. OR (95% CI)	*P*-value
Chief complaint		
Gastrointestinal bleeding	0.12 (0.01, 1.35)	0.066
Abdominal mass	1.76 (0.13, 24.12)	
Early satiety, vomiting	0.23 (0.02, 2.22)	
Accidental findings	Ref.	
Intraoperative perforation	0.05 (0, 0.69)	0.026
High risk category	27.67 (4.29, 178.63)	<0.001
Exposed imatinib	3.15 (0.73, 13.66)	0.125

Adj, adjust; OR; odd ratio; Ref, reference.

**Table 5:** Determination of factors predicting disease free survival in patients with gastric GIST using a backward stepwise logistic model.

Variables	Adj. HR (95% CI)	*P*-value
Age ≥60 years	0.01 (0, 0.39)	<0.001
Female	48.38 (1.12, 2,085.44)	0.110
History of smoking	706.18 (3.59, 139,005.75)	<0.001
Staging		
1	Ref.	<0.001
2	25.92 (0.76, 879.04)	
3	1,850.47 (13.99, 244,835.05)	
4	2,377.18 (12.88, 438,866.44)	
History of preoperative biopsy	48.87 (1.83, 1,303.09)	0.003
Operative time ≥200 min	13.15 (0.85, 203.42)	0.034

Adj, adjust; HR; hazard ratio; Ref, reference.

These findings highlight the necessity of aggressive disease management, early intervention, and comprehensive perioperative care strategies to improve survival outcomes in gastric GIST patients.

## Discussion

This study provides an in-depth evaluation of prognostic factors influencing survival outcomes in gastric GIST patients. Our findings confirm that high-risk classification, larger tumor size, and higher mitotic rates are key determinants of worse DFS and increased mortality. Compared to previous studies, our cohort exhibited similar prognostic trends, reinforcing the importance of aggressive disease management in high-risk patients (11, 12). However, our findings also shed light on unique aspects of disease progression and treatment responses, particularly in resource-limited settings.

One of the most striking findings is the significant disparity in DFS between high-risk and non-high-risk patients. The 5-year DFS rate for high-risk patients was considerably lower (40% vs. 95.7%, *p* < 0.001), consistent with prior studies. However, our study did not find a significant difference in OS between high-risk and non-high risk groups. This could be influenced by the administration of imatinib therapy during disease progression, potentially prolonging survival even after recurrence. Unfortunately, due to the limitations of our dataset, we lack precise information on the number of patients treated with imatinib at progression, making this an assumption rather than a confirmed conclusion. This highlights the need for future studies with detailed treatment tracking to better assess the impact of post-recurrence imatinib therapy on OS (13). Besides, in our study, only 5 patients (5.8%) received preoperative imatinib, which is lower than the number of stage IV patients (*n* = 6, 7%). This limited use reflects real-world clinical practice, where preoperative imatinib is typically reserved for locally advanced or borderline-resectable tumors rather than standard treatment for all high-risk cases. Due to the small sample size, we were unable to evaluate the impact of preoperative imatinib on DFS and OS, highlighting the need for future prospective studies assessing its potential role in gastric GIST management.

Another unexpected finding was that tumor perforation (capsule rupture) did not significantly impact DFS. While previous studies have consistently reported tumor perforation as an unfavorable prognostic factor, our results suggest that aggressive postoperative management and surveillance may have mitigated its negative impact. Interestingly, multivariate analysis indicated that tumor perforation was associated with a statistically significant reduction in mortality (HR 0.05, *p* = 0.026) which contradicts existing literature. This result could be due to selection bias, sample size limitations, or differences in how tumor perforation was classifiedrupture (14). Future studies are needed to explore whether standardized postoperative monitoring protocols can improve long-term outcomes in these patients.

Imatinib remains the cornerstone of adjuvant therapy for high-risk GIST patients, yet our findings reveal an unexpected association between imatinib exposure and worse DFS (*p* = 0.003). This paradoxical finding likely reflects delayed initiation of imatinib in our cohort, where most patients received the drug only after recurrence or metastasis rather than as true adjuvant therapy. Previous studies have demonstrated that timely administration of imatinib significantly improves survival outcomes, but in our setting, restrictions on drug accessibility may have contributed to suboptimal treatment outcomes (15). These findings underscore the importance of policy adjustments to ensure that high-risk patients receive early and sustained imatinib therapy to maximize its therapeutic benefits.

Our study also found that tumor size (>10 cm) and mitotic index (>5 per 50 HPF), despite being included in all major risk stratification models, were not statistically significant prognostic factors for DFS in our cohort. This contradicts well-established literature but may be explained by the relatively small sample size, heterogeneity in treatment patterns, and potential confounding by other strong prognostic variables such as resection status and postoperative therapy (12, 13). Similarly, R1/R2 resection status, which is widely recognized as an indicator of poor prognosis, did not significantly affect DFS in our analysis. The low proportion of R1/R2 cases in our study (11.6%) and the potential mitigating effect of adjuvant therapy or close follow-up could explain this unexpected result (14). Future larger-scale studies are needed to validate these findings. Another significant prognostic factor identified in our study is smoking, which was strongly associated with worse DFS (HR 706.18, *p* < 0.001). While the detrimental effects of smoking are well-documented in various malignancies, its impact on GIST outcomes has been underreported (16). Our study highlights the need for integrating smoking cessation programs into perioperative management protocols for GIST patients. Further research is needed to elucidate the underlying mechanisms by which smoking contributes to disease recurrence and whether interventions aimed at modifying this risk factor can improve DFS (17).

Our study also provides new insights into the impact of prolonged operative time (≥200 min) on DFS (HR 13.15, *p* = 0.034). This finding suggests that more complex surgical cases, requiring extended operative durations, may be associated with greater tumor manipulation, increased morbidity, and higher recurrence rates. This observation warrants further investigation into the role of minimally invasive surgical techniques in improving DFS outcomes, particularly in high-risk patients requiring extensive resections (18).

One of the key knowledge gaps addressed by this study is the real-world impact of treatment limitations in resource-constrained settings. Unlike studies conducted in high-income countries where adjuvant imatinib is widely accessible, our results illustrate the detrimental effects of delayed imatinib initiation on DFS (19). This finding represents a major contribution to the existing literature and provides compelling evidence for policy changes aimed at improving early access to targeted therapy for high-risk patients. By highlighting the challenges associated with treatment accessibility, our study serves as an advocacy tool for expanding the availability of imatinib and other TKIs in low- and middle-income countries.

Additionally, while previous studies have extensively examined tumor characteristics and surgical factors, the influence of lifestyle factors such as smoking on DFS has remained largely unexplored (20). Our study addresses this gap by demonstrating a clear association between smoking and disease recurrence, suggesting that lifestyle modifications should be incorporated into standard GIST treatment protocols. Further prospective studies are needed to validate these findings and assess the impact of smoking cessation on long-term survival outcomes in GIST patients.

The strengths of this study include its comprehensive multivariate analysis, which accounts for multiple confounding factors, and its emphasis on real-world clinical practice in a developing country setting. However, limitations must also be acknowledged. The retrospective nature of the study introduces potential selection bias, and the relatively small sample size may limit the generalizability of our findings. Additionally, we lacked detailed data on the timing and duration of imatinib therapy after recurrence, which could have provided further insights into its impact on OS. Future prospective trials with larger cohorts and longer follow-up periods are warranted to validate our observations and further investigate the optimal timing of imatinib initiation.

## Conclusion

This study highlights significant differences in survival outcomes between high-risk and non-high-risk gastric GIST patients. High-risk classification, larger tumor size, and higher mitotic rate were strongly associated with worse DFS and increased mortality. Despite the established benefits of adjuvant imatinib, our findings indicate that patients who received imatinib exhibited worse DFS outcomes, likely due to delayed therapy initiation. These findings emphasize the necessity of improving access to timely imatinib therapy and closer postoperative monitoring, particularly in high-risk patients, to optimize long-term outcomes. Additionally, the strong association between smoking and DFS underscores the need for lifestyle modifications as part of comprehensive GIST management strategies. Our findings serve as an important step toward advocating for policy changes to improve the accessibility of targeted therapy and reinforce the need for enhanced perioperative management strategies in high-risk gastric GIST patients.

## Data Availability

The original contributions presented in the study are included in the article/Supplementary Material, further inquiries can be directed to the corresponding author.
